# Characterization of FFPE-induced bacterial DNA damage and development of a repair method

**DOI:** 10.1093/biomethods/bpaa015

**Published:** 2020-07-27

**Authors:** Yensi Flores Bueso, Sidney P Walker, Mark Tangney

**Affiliations:** b1 CancerResearch@UCC, University College Cork, Cork, T12 XF62, Ireland; b2 SynBioCentre, University College Cork, Cork, T12 XF62, Ireland; b3 APC Microbiome Ireland, University College Cork, Cork, T12 YT20, Ireland

**Keywords:** base excision repair, DNA damage, genomics, metagenomics, formalin-fixed paraffin-embedded

## Abstract

Formalin-fixed, paraffin-embedded (FFPE) specimens have huge potential as source material in the field of human microbiome research. However, the effects of FFPE processing on bacterial DNA remain uncharacterized. Any effects are relevant for microbiome studies, where DNA template is often minimal and sequences studied are not limited to one genome. As such, we aimed to both characterize this FFPE-induced bacterial DNA damage and develop strategies to reduce and repair this damage. Our analyses indicate that bacterial FFPE DNA is highly fragmented, a poor template for PCR, crosslinked and bears sequence artefacts derived predominantly from oxidative DNA damage. Two strategies to reduce this damage were devised – an optimized decrosslinking procedure reducing sequence artefacts generated by high-temperature incubation, and secondly, an *in vitro* reconstitution of the base excision repair pathway. As evidenced by whole genome sequencing, treatment with these strategies significantly increased fragment length, reduced the appearance of sequence artefacts and improved the sequencing readability of bacterial and mammalian FFPE DNA. This study provides a new understanding of the condition of bacterial DNA in FFPE specimens and how this impacts downstream analyses, in addition to a strategy to improve the sequencing quality of bacterial and possibly mammalian FFPE DNA.

## Introduction

Formalin-fixed, paraffin-embedded (FFPE) samples represent the most comprehensive collections of patient materials in hospital pathology archives [1–3]. These samples can provide access to bacterial communities inhabiting a variety of body sites for which access to ‘fresh’ tissue samples is limited [4, 5] due to the invasive nature of their sampling [6–11]. However, as has been definitively shown from analysis of human DNA [12], FFPE processing induces DNA damage. In mammalian DNA, this damage occurs as: (i) cross-links (DNA–DNA, Protein–DNA) [13, 14], (ii) depurination [15–17], (iii) DNA fragmentation [18, 19] and (iv) sequence alterations [chimeras, single nucleotide polymorphisms (SNPs)] [20, 21], which accumulate further with storage time and suboptimal fixing conditions [12, 22]. This DNA damage has been found to negatively affect mammalian DNA sequencing outputs, by reducing: (i) the sequencing depth, (ii) sequencing uniformity, (iii) read length, (iv) ratio of reads passing quality filtering; and increasing (i) the number of chimeric reads, (ii) FFPE-derived SNPs, translocations, and insertions and deletions (indels) [12, 23–28].

Bacterial DNA is likely to be similarly damaged, but this is uncharacterized to date. The consequence of such bacterial DNA damage is that FFPE samples will have several associated limitations that must be considered before their effective use in microbiome studies. DNA fragmentation reduces the quantity of DNA fragments within a sample of suitable length for amplicon-based sequencing strategies such as 16S rRNA gene sequencing (∼460 bp for V3-V4 [29]). This can exacerbate the characteristic low-bacterial biomass found in FFPE samples. FFPE-induced sequence alterations can decrease sequence quality and lead to false speciation events. These are considerable hurdles standing in the way of accurate, reproducible microbiome research from FFPE samples. All research reported to date, and protocols for purifying and repairing FFPE DNA, relate to mammalian (human) DNA. Differences in DNA conformation and packaging, methylation patterns, and replication and transcription rates, between human and bacteria may lead to different FFPE damage profiles [30–32]. A better understanding of potential differences is essential for the proper design of workflows that ensure bacterial DNA quality and guarantee reliable and reproducible sequencing analysis [33]. No characterization of FFPE-induced bacterial DNA damage exists to date.

The product of the interaction between formaldehyde and biomolecules is the formation of crosslinks (methylene bridges). These are ubiquitous in FFPE specimens, where they are more frequently found as DNA–protein crosslinks (DPC) between dG and amino acids Lysine and Cysteine [34, 35]. These crosslinks strain the DNA structure, promoting apurinic (AP) sites and ss-breaks [16, 36] and inhibiting polymerase chain reaction (PCR)[37]. Fortunately, crosslinks are reversible, the intermediary products (Schiff bases) are reversed by hydration [35] and methylene bridges are reversed with heat treatment [13]. As such, all protocols for FFPE DNA purification available incorporate a heat treatment decrosslinking step, typically as 1 h incubation at 90°C [14]. However, recent studies have shown that this high incubation temperature increases the frequency of ss-breaks and chimeric sequences. A lower incubation temperature reduces the appearance of these sequence artefacts, but with the caveat of reducing the total yield of decrosslinked DNA [20, 21]. This suggests that there is potential for an optimized decrosslinking strategy allowing for reduced incubation temperature, without diminishing the yields of decrosslinked DNA.

Assuming the existence of sequence artefacts (AP sites, damaged bases, ss-breaks), the base excision repair (BER) pathway, the main cellular pathway for their repair in metabolically active cells, represents a promising opportunity for their repair [38, 39]. Indeed, improvement of the sequencing quality of FFPE specimens has been attempted using DNA glycosylases (uracil-DNA glycosylase) [40]. In addition, commercial kits for some degree of FFPE DNA repair have recently become available: ‘NEB FFPE DNA Repair’ and ‘Illumina Infinium FFPE Repair’; however, their composition is proprietary and undisclosed. Despite such advances, there is a gap in the literature characterizing DNA damage recognition by DNA glycosylases on FFPE specimens, which is essential for designing approaches to reconstitute the BER pathway to repair FFPE DNA damage [41]. The BER pathway can be summarized in five steps. (i) Base excision by a DNA glycosylase, followed by (ii) backbone excision by an AP lyase or AP endonuclease, (iii) ends processing by a polynucleotide kinase (PNK) or exonuclease, (iv) gap filling by a polymerase and (v) nick ligation by a ligase [38, 39]. The type of DNA glycosylase determines downstream repair workflow. Monofunctional DNA glycosylases (i.e. UDG), yield an AP site that is excised by an AP endonuclease (i.e. Endo IV), generating a nick with deoxyribose phosphate (dRP) residue in the 5′ terminus and a clean 3′OH terminus. This 5′dRP residue is removed by the 3′–5′ exonuclease activity of DNA polymerase (long-patch BER). On the contrary, bifunctional glycosylases (i.e. those repairing oxidative damage), can cleave the backbone by β- or β-δ-elimination. The product of β-elimination is a phospho-α,β-unsaturated aldehyde that can be removed by an AP endonuclease, and repaired as monofunctional glycosylases. The product of β/δ-elimination is a 3′ end phosphate that is removed by a PNK and the lesion filled through short-patch BER [38, 39, 42–46].

In this study, a ‘mock’ FFPE model replicating the conditions found in clinical FFPE samples was used to characterize the nature and severity of FFPE-induced damage in bacterial DNA, followed by development of an effective strategy for repairing it. Quantitative PCR and high-resolution melt (HRM) analysis, along with Sanger sequencing were used to screen decrosslinking conditions and available DNA glycosylases, shortlist those found most effective. These were then further tested individually and in combination, with a final validation of whole genome sequencing (WGS) analysis used to determine the most effective DNA repair strategy.

## Material and methods

### Preparation of FFPE blocks

#### Bacterial growth conditions

*Escherichia coli* K12 MG1655 or *E. coli* Nissle 1917 carrying a P16Lux plasmid [47] was grown aerobically at 37°C in Luria-Bertani (LB) medium with 300 µg/ml Erythromycin (Sigma-Aldrich). *Staphylococcus aureus* Newman (ATCC 25904) was grown aerobically at 37°C in Todd-Hewitt broth (Sigma-Aldrich). *Bifidobacterium longum* has grown anaerobically at 37°C for 24 h in De Man, Ragosa, Sharpe (MRS) medium (Sigma-Aldrich). *Lactobacillus amylophilus* (ATCC^®^ 49845^™^) was grown in MRS medium (Sigma-Aldrich) at 30°C in 5% CO2 for 24 h. *Bacteroides thetaiotaomicron* (ATCC^®^29741^™^) was grown anaerobically at 37°C for 24 h in Fastidious Anaerobe Broth (FAB) medium (NEOGEN, Lancashire, UK). Bacterial cultures were harvested by centrifugation and suspended in PBS. A 1 ml aliquot of the suspension was used to count colony-forming units (CFUs) by retrospective plating. The rest was resuspended in neutral buffered formalin and left to fix for 18 h at room temperature (RT).

#### Counting fixed bacterial cells

The cell suspension was counted using a bacterial counting kit for flow cytometry (Invitrogen). In brief, a 10% aliquot from the bacterial suspension was serially diluted to 1 × 10^6^ cells in 989 µl of NaCl. Bacterial cells were stained with 1 µl of SytoBC and 10 µl (1 × 10^6^) of counting beads were added to the suspension. Cells were counted in an LSR II flow cytometer (BD Biosciences). The acquisition trigger was set to side scatter and regulated for each bacterial strain to filter out electronic noise without missing bacterial cells. This value was ∼800. The volume corresponding to ∼2 × 10^7^ CFU of each bacterial strain and 2.2 × 10^7^ 4T1 cells were mixed together.

#### Cell culture

*Mus musculus* mammary gland cancer cells (4T1) were grown at 37°C 5% CO_2_, in RPMI-1640 (Sigma-Aldrich) media supplemented with 10% FBS (Sigma-Aldrich), 100 U/ml penicillin and 100 μg/ml of streptomycin (Thermo Fisher) and counted with a NucleoCounter^®^ NC-100^™^ (Chemometec, Copenhagen).

#### Fixing cells in an agar matrix

An equal volume of sterile agar (1.5× of elution specified by the manufacturers) prealiquoted and kept at 56°C was pipetted into the cell suspension and thoroughly mixed by vortexing. The mixture was pipetted into a sterile cylindrical mould made from a 54 mm × 11 mm adapter tube (Sarstedt, Cat No. 55.1570) and let solidify for 3 min. Once solidified, the disk was placed in 5 ml of formalin for an extra 24 h for 48 h fixation blocks or immediately processed for 24 h fixation blocks. These are termed ‘Protoblocks’.

#### Dehydration, paraffin embedding of cell disk and sectioning

Fixed cell disks were removed from the formalin and placed into a processing cassette. The cassettes containing the Protoblocks were dehydrated and paraffin-embedded automatically with a LOGOS J (Milestone Medical, Bergamo). This protocol included 4 h dehydration with increasing concentrations of ethanol, clearing with ×2 washes of xylene and ×3 washes of isopropanol. Finally, the blocks were embedded in paraffin for 8 h and 32 min at 62°C. Once paraffinized, the Protoblocks’ volume, diameter and height were measured with a calliper and by volume displacement [48]. Processed Protoblocks were placed in a 1.5 cm × 1.5 cm embedding mould and mounted to a processing cassette. Blocks were sectioned keeping an aseptic technique either at 4 µm for imaging or at 15 µm for DNA purification. The cell load of each slide was calculated by dividing the total bacterial load by the volume of each slide.

#### Immunofluorescence and histochemistry

Cell morphology was evaluated with Gram staining (Sigma-Aldrich) or H&E staining with Mayer’s haematoxylin (Sigma-Aldrich). Bacterial counts were confirmed in three sections stained with DAPI, 1:50 α-*E. coli* (Abcam, 137967), or 1:400 α-*S. aureus* (Abcam, 20920), and counterstained with either Alexa Fluor 488 (Jackson Immunoresearch Laboratories Inc., USA) donkey anti-rabbit Ig. Stained sections were mounted in ProLong Gold Antifade reagent with DAPI (Invitrogen, UK). Gram-stained sections were counted in bright field using an Olympus BX51 microscope, with a ×100 lens. Immunofluorescent stained slides were counted at ×20 (4T1 cells) or ×60 (bacteria) with a fluorescence microscope (Evos FL Auto). For each slide, at least 20 randomly selected fields of view were counted. The area of the field of view was recorded using the microscope’s software and used to calculate the volume counted.

### DNA analysis

#### DNA purification

For purifying DNA from Protoblocks, unless specified, 10 μm × 15 μm sections aseptically collected sections were deparaffinized with ×2 xylene washes and processed following procedures specified in the QIAGEN FFPE DNA kit protocol (Qiagen Inc., Valencia, CA, USA). DNA was eluted in Tris-HCl (pH 8) and quantified with a Qubit^™^ dsDNA HS Assay Kit (Invitrogen, USA). For non-fixed (NF) bacteria, bacterial cultures were grown to an OD_600_ of 1. 2 ml aliquots were processed following procedures of the GenElute^™^ Bacterial Genomic DNA Kit Protocol with lysozyme and lysostaphin (Sigma) and eluted in 50 µl of Tris-HCl (pH 8). In all cases, DNA was stored at −20°C until further analysis.

#### Quantitative PCR

For quantitative qPCR, reactions were prepared using LUNA Universal qPCR (NEB, Ipswich, MA, USA) and 0.25 µM of each primer (Supplementary Table S1). The thermal profile included an initial denaturation of 1 min at 95°C, and 40 cycles of denaturation at 95°C for 10 s, annealing for 15 s at the primers’ optimal temperature (54°C–56°C) (specified by New England Biolabs (NEB) calculator for Hot Start *Taq*) and 20–40 s of extension at 68°C (20 s for 200 bp amplicons and 40 s for 400–500 bp amplicons).

#### High-fidelity quantitative PCR reaction set-up

Reactions were prepared using NEBNext-Ultra II Q5 Master Mix, 0.5 µM of each primer (Supplementary Table S1), 1.25 µM EvaGreen Dye (Biotium, CA, USA) and 37.5 nM ROX (Biotium, CA, USA) as a reference dye. The thermal profile included an initial denaturation of 30 s at 98°C, and 40 cycles of denaturation at 98°C for 10 s, annealing for 15 s at the primers’ optimal temperature (64°C–67°C) (specified by NEB’s calculator for Q5 High-Fidelity Master Mix) and 20–40 s of extension at 72°C (20 s for 100–200 bp amplicons and 40 s for 400–500 bp amplicons).

#### Quantitative qPCR assays parameters

Amplification was performed in an AriaMx (Agilent Technologies, USA) using DNA-binding dye absolute quantitation experiment type. Each assay included triplicates of 5 points standards using log-dilutions of a 10^7^ copies gene block, designed upon a species-specific genetic region. Primers targeting these regions and maintaining a similar Tm (±2°C) were designed using the National Center for Biotechnology Information (NCBI) primer design tool and their parameters (ΔG, hairpins and dimers) verified using IDT’s oligo analyser tool. Primers and gene-blocks were acquired from IDT (Coralville, USA) (see Supplementary Table S1). The qPCR efficiencies between 95% and 105% and *R*^2^ values >0.995 were deemed as acceptable, all samples were run in triplicate.

#### High-resolution melt (HRM) curve analysis

For melt curve analysis, it was essential to first normalize the amplifiable DNA fraction of samples tested. To achieve this, a quantitative qPCR was performed for fragments of the same length. The measured copy numbers obtained by qPCR were used to normalize the samples to 1 × 10^6^ copies/µl. The 20 µl reactions were prepared using ×1 NEB Luna probe qPCR mix, 1.25 µM EvaGreen Dye (Biotium, CA, USA), 37.5 nM ROX as reference dye, 0.25 µM of each primer and 2.5 µl of copy number normalized template DNA. *Escherichia coli* primers rendering amplicons of 100, 200 and 500 bp were used for this assay (Supplementary Table S1). The amplification of the analysed target region was first amplified as specified for absolute quantitation, but included a final 2 min at 68°C extension step. This was followed by HRM analysis set to read fluorescence every 0.2°C with a 10 s soak time from 65°C to 95°C. All experiments were performed using an AriaMx thermocycler (Agilent Technologies).

Here, normalized fluorescence (Rn) obtained every 0.2°C, across the temperature gradient (65°C–95°C), was used to monitor the melting temperature (Tm) profile of the template. Changes in the Tm profile are indicative of changes in the template sequence. To better observe these changes, the Tm profiles were plotted on a Tm difference (ΔTm) plot, where the Tm difference is represented by the deviation of the recorded Rn values of a test plotted against those recorded for a NF reference, for which the ΔTm is 0. Therefore, ΔTm = Rn Test – Rn of reference. Here where aberrant profiles that differ from NF DNA with ΔTm < 0.1°C are typical of FFPE DNA and are indicative of low-level, non-identical changes randomly distributed across the template [49]. Therefore, in these plots, a lower ΔTm is indicative of a reduced/lower number of sequence artefacts in the template. Raw Tm values were extracted from the AriaMx software and analysed in R environment, v3.4.4.

#### Sanger sequencing

Sanger sequencing was performed on 500 ng of purified and/or treated DNA for each replicate on the same genomic regions analysed by qPCR. Sequencing was performed by Eurofins Genomics.

#### WGS sequencing library preparation

For NF controls, DNA from bacterial cultures of *E. coli* MG1655 and *S. aureus* Newman were grown as per section 1 and OD_600_ of 1 and their genomic DNA purified using the GenElute^™^ Bacterial Genomic DNA Kit Protocol with Lysozyme and Lysostaphin (Sigma). For FFPE bacteria, DNA from Protoblocks containing either strain was purified using the QIAGEN FFPE kit plus specified treatment. In all cases, DNA was eluted in 50 µl of Tris-HCl (pH 8). Total purified DNA and/or repaired DNA was sent to GENEWIZ (Leipzig, Germany) where WGS was performed using 2 × 150 bp chemistry on an Illumina HiSeq.

### Optimizing cross-link reversal

#### Temperature-point experiments

The 10 µm × 15 µm sections from blocks loaded with 10^8^*E. coli* and *S. aureus* cells fixed for 24 h and stored for 3 months were distributed into 12 ml × 1.5 ml tubes. The deparaffinized and digested contents were pooled and distributed into 24 experimental replicates, 6 replicates per temperature point tested (90°C, 80°C, 72°C and 65°C). For temperature points 90°C and 80°C, incubation time was set for 1 h and for 72°C and 65°C it was set for 2 h. After decrosslinking, the DNA content was purified with the QIAGEN FFPE protocol.

#### Cross-link reversal buffer

Lysis buffers tested for cross-link reversal were TB1 (50 mM Tris-HCL (pH 8.0), 30 mM EDTA, 800 mM guanidine hydrochloride (GuHCl) , 0.5% Triton-X, 0.5% Tween-20), TB2 [50 mM Tris-HCl (pH 8.2), 100 mM NaCl, 1 mM EDTA, 0.5% Tween-20, 0.5% NP40, 20 mM Dithiothreitol (DTT)] and TB3 [50 mM Tris-HCl (pH 8.0), 100 mM EDTA (pH 8.0), 100 mM NaCl, 1% SDS]. The 10 µm × 15 µm slides from blocks loaded with 10^8^*E. coli* and *S. aureus* cells fixed for 24 h and stored for 3 months were used per experimental replicate (six per buffer tested). The samples were lysed and digested in the experimental buffer at 56°C for 1 h and decrosslinked at 80°C for 1 h. After testing for decrosslinking buffers, an equal volume of buffer AL (column binding buffer) was added to the reaction and the DNA content purified following the QIAGEN FFPE kit protocol.

#### Verifying cross-link reversal strategy

A total of 10 µm × 15 µm slides from blocks loaded with 10^8^*E. coli* cells fixed for 48 h and stored for 1 year were used per experimental replicate (six per test). After decrosslinking, the DNA content purified with the QIAGEN FFPE kit.

### DNA repair

#### Treatment with individual glycosylases

DNA purified from FFPE blocks loaded with 10^8^*E. coli* cells fixed for 24 h or 48 h was pooled and its concentration measured and normalized across tests. Aliquots with equal DNA concentration were used for each experimental replicate. All enzymes tested were acquired from NEB (Ipswich, MA, USA) and the verified enzyme activity provided by the supplier used to calculate the amount of enzyme input. Genomic data shown in Supplementary Table S2 was used to calculate the enzyme input per nanogram of *E. coli* K12 MG1655 DNA. For this, enzymatic activity was first normalized in terms of number of damaged nucleotides or lesions repaired by an enzyme unit in a standard 30 min reaction. An estimate of 0.05–0.1% of damaged bases in FFPE DNA was used as a baseline. With this information, the number of damaged bases was first calculated per nanogram of DNA in the reaction and the enzyme units required to repair this damage. The units of enzyme used were optimized to fit the activity in a universal buffer and after titration experiments. The final units used in the reaction and the number of bases corrected per nanogram of *E. coli* DNA are listed in Supplementary Table S3. The 40 µl reactions were set-up using a total of 400–1,000 ng of bacterial DNA. The reactions were run at 37°C for 30 min, after which enzymes were heat-inactivated with incubations specified in Supplementary Table S3. Treated DNA was cleaned using the Monarch PCR & DNA Clean-up Kit (NEB, USA). DNA concentration was measured with QUBIT (Invitrogen) and normalized DNA quantities analysed by quantitative PCR or HRM.

### Assembling BER reaction

#### Buffer

The BER pathway was reconstituted in a final buffer with 1X NEB CutSmart buffer (50 mM potassium acetate, 20 mM Tris-Acetate, 10 mM Magnesium acetate and 100 µg/ml of bovine serum albumin, pH 7.9), supplemented with 100 µM of dNTPs, 50 µM of NAD+ and 2 mM of DTT. Enzyme efficiency in this buffer was analysed by comparing its activity with the buffer provided by the manufacturer. The compared enzyme activity was used to adjust the enzyme units used for the BER reaction.

#### Repair of excised bases

The repair of excised bases was accomplished with long (UDG) and short patch BER (Formamidopyramidine DNA glycosylase (FPG), Endo VIII), by incorporating the downstream enzymes that repair blocked ends (PNK) or AP sites (Endo IV), plus DNA polymerase I and *E. coli* DNA ligase (Fig. 4 and Supplementary Table S4). DNA glycosylases tested were UDG, FPG and Endo VIII. Enzymes were acquired from NEB (Ipswich, MA, USA). The reactions were prepared with the buffer described in the buffer section, using normalized DNA quantities and carried out at 37°C for 30 min. The reactions were stopped with the addition of 2X volumes of Agencourt AMPure XP magnetic beads (Beckman Coulter, IN, USA) for DNA clean up. Following the manufacturer’s instructions, DNA was washed twice with 80% ethanol and eluted in 36 µl of Tris-HCl (pH 8). DNA concentration was again measured for each reaction and normalized DNA quantities were used for quantitative PCR, HRM, or by Sanger sequencing.

#### BER with combined glycosylases

These reactions were set-up and carried out as described above for repair of excised bases, with the difference that in these reactions these were combined and included the combined downstream lesion repair enzymes (Endo IV and PNK) specified for the sub-pathway triggered. Here, for mixes containing UDG, Endo IV was included and form mixes containing FPG or Endo VIII, PNK was included. All mixes included DNA Pol I and DNA Ligase. All enzymes were acquired from NEB (Ipswich, MA, USA). The reactions were analysed by HRM, Sanger sequencing and WGS.

### Bioinformatics and statistical analysis

#### The qPCR and HRM data analysis

Statistical analysis performed in the base R environment (v3.6.1). Visualizations were carried out using the ggplot2 package (v3.2.1).

#### Sanger sequence analysis

The effect of DNA repair enzymes on DNA sequence length and readability was assessed by Sanger sequencing. The ratio of clipped sequence length to unclipped sequence length between samples was compared to elucidate this. Statistical analysis performed in the base R environment (v3.6.1). Visualizations were carried out using the ggplot2 package (v3.2.1).

#### WGS sequence analysis

All metrics relating to sequence data were calculated in the Linux environment, and using the QUAST tool (v5.0.2) and statistical analysis performed in the base R environment (v3.6.1). Visualizations were carried out using the ggplot2 package.

### Method for variant calling

#### Filtering

HiSeq sequence data were quality filtered. Only very high-quality bases (Phred score >30) were considered to minimize the risk of sequencing errors causing false-positive variants. Short fragments were also removed to reduce the likelihood of spurious alignments of regions from contaminant bacterial genomes. Trimmomatic (v0.38) was used to remove all reads shorter than 60 bp in length, and to trim reads when the average per base quality in a sliding window of size 4 dropped below 30.

#### Alignment

Of the three possible Burrows–Wheeler alignment tools, the BWA-mem aligner was used as the average read length was 150 bp, and BWA-mem (v0.7.17) is recommended when reads are over 70 bp in length. Default settings were used with the exception of allowing alignments with a minimum score of 0, rather than the default 30. Given the stringent parameters used for read length and quality filtering, relaxing the minimum alignment score gave the best possible chance of variant detection. All samples were aligned with the original reference genomes.

#### Variant calling

Variant calling was done with BCF tools, using the BCF call function. The variants were then filtered using the norm and filter functions within BCF tools. Filtering was done to remove variants when the read depth was below 10, the quality was below 40, or when the variant identified was not supported by both the forward and reverse read of a read pair. The number of variants identified was then normalized between samples based on the read coverage in the initial alignment BAM file.

#### Validation

Using the Picard tool within the Genome Analysis Tool Kit suite, all samples were down-sampled to ensure SNP: coverage ratio remained constant when coverage was reduced to lowest level present in samples.

## Results

### Characterization of bacterial FFPE DNA damage

#### Measuring fragmentation of PCR readable DNA

**Figure 1:**
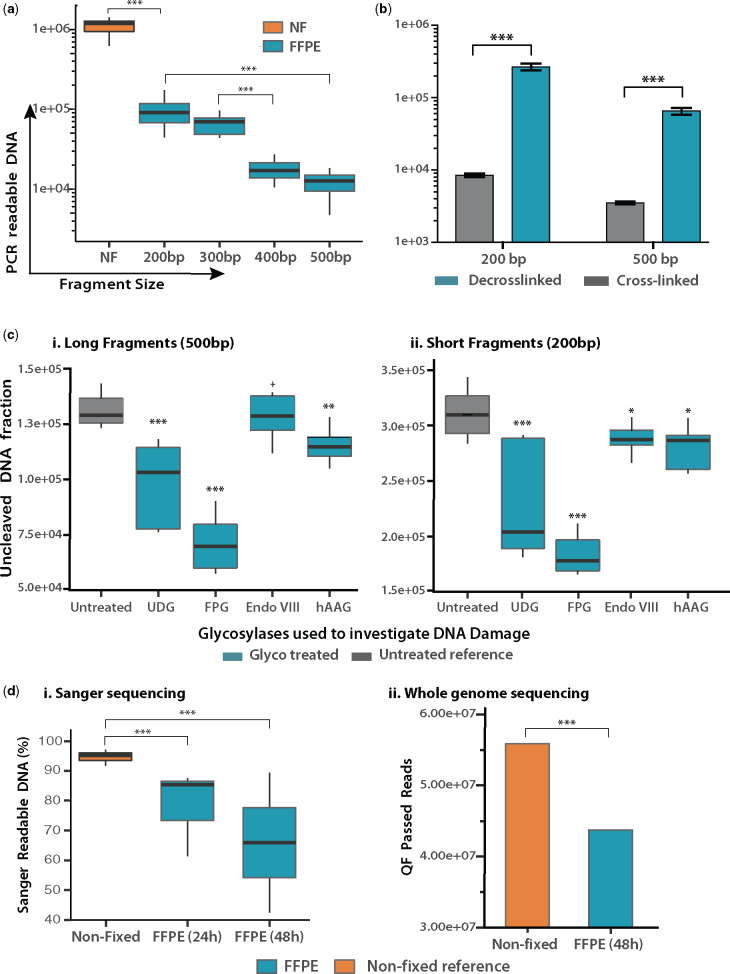
Analysis of bacterial FFPE DNA damage. (**a**) Measuring fragmentation of PCR-amplifiable DNA. For NF bacteria, amplification of all fragment lengths was equal and grouped in the same box (*n* = 28). For FFPE bacteria (*n* = 24 for each box), a linear fragment-length correlation is evident, with a log-decrease observed from NF to FFPE 200 bp fragments and a log-decrease between short (200 bp) and long (500 bp) fragments (*P* < 0.001). (**b**) Assessing the extent of cross-links in bacterial DNA. DNA from FFPE blocks containing *E. coli* cells was subjected (*n* = 6) or not (*n* = 6) to a high-temperature cross-link reversal treatment. The bar plot shows the quantity of amplifiable DNA obtained ± cross-link reversal for long and short DNA fragments. Without decrosslinking, only 3–5% of the available DNA template is amplifiable for PCR. (**c**) Evaluating the presence of damaged nucleotides via glycosylase treatment. Box plots show the quantity of amplifiable DNA post-treatment with the respective glycosylase (*n* = 6 in all cases). (**d**) Assessment of DNA sequence quality by sequencing. (i) Sanger sequencing showing the percentage DNA falling within the high confidence read region for each sample. (ii) WGS showing the number of QF pass reads for FFPE and NF bacteria.

The length of PCR-readable fragments from bacterial DNA subjected to FFPE treatment was measured by quantitative PCR. Targeting a 525 bp chromosomal region, primers were designed to amplify DNA fragments of lengths 200 bp, 300 bp, 400 bp and 500 bp. Template DNA was purified from FFPE blocks loaded with 1 × 10^8^*E. coli* cells, fixed for 48 h and stored for >6 months. Each qPCR reaction was loaded with 5 ng of DNA, corresponding to 1 × 10^6^ CFU. As seen in Fig. 1a, the quantity of amplifiable DNA is significantly reduced after FFPE treatment. For NF DNA, the amplification of PCR-readable fragments is almost 100% and is independent of fragment size, whereas a log-fold reduction of amplifiable DNA is observed for even short (200 bp) fragments of FFPE DNA (*P* < 0.001). This becomes more pronounced as fragment length increases, with significant correlation between reduction in the quantity of amplifiable DNA and fragment length, leading to a log-fold reduction in amplifiable DNA quantity between 200 bp and 500 bp fragments (*P* < 0.001).

#### Assessing the extent of formaldehyde cross-links in FFPE bacterial DNA

The presence and frequency of formaldehyde crosslinks present in bacterial DNA were assessed by comparing the quantity of amplifiable DNA obtained after performing or omitting a cross-link reversal incubation on paired-samples (*n* = 6), a strategy resembling the straightforward formaledyhe assisted isolation of regulatory elements (FAIRE) method [13]. As can be seen in Fig. 1b, crosslinking was evident regardless of fragment size, with an 18.5 (500 bp) – 30 (200 bp) fold increase in amplifiable DNA observed after cross-link reversal, indicating that 95–97% of the amplifiable DNA in the sample held crosslinks that inhibited its amplification.

#### Evaluating the presence of damaged nucleotides

The presence of damaged bases in bacterial FFPE DNA was investigated by subjecting FFPE-DNA to the activity of DNA glycosylases targeting base oxidation, deamination and carboxylation with enzymes listed in Supplementary Table S3. DNA lesions resulting from DNA glycosylase activity (AP sites and 3′P) [39, 43], inhibit amplification [50]. Therefore, DNA glycosylase activity can be measured by comparing the quantity of amplifiable DNA in a sample after treatment/no treatment with a DNA glycosylase, with a decrease in amplification implying the presence of the targeted DNA damage. As seen in Fig. 1c, a decrease in amplifiable DNA was noticeable in concentration normalized samples after treatment with all glycosylases, with the highest activity observed for UDG and FPG as indicated by the 35–50% and 67–80% reduction in the recovery of PCR readable DNA fragments after treatment (*P* < 0.001) (Fig. 1c). It should be noted that Endo VIII activity is not measurable by this PCR analysis, as lesions targeted by this enzyme (hydantoins) are PCR inhibitory, thus, the removal of this damage would not have any effect on the amount of amplifiable DNA template [51].

#### Assessment of DNA sequence quality by sequencing

Overall DNA damage is reflected in the outputs of sequencing. Damaged bases and single-strand breaks present as sequencing misreads, such as chimeras, indels and SNPs that lead to poor quality reads, which will be routinely filtered out prior to analysis. As seen in Fig. 1d, a significant decrease in high-quality, sequencing-readable DNA was observed in both Sanger sequencing and WGS, for FFPE samples compared with their paired NF samples. This was accentuated by prolonged DNA fixation, where the reduction of high-quality sequences reaches 30% (*P* < 0.001).

### Development of a DNA repair strategy

**Figure 2:**
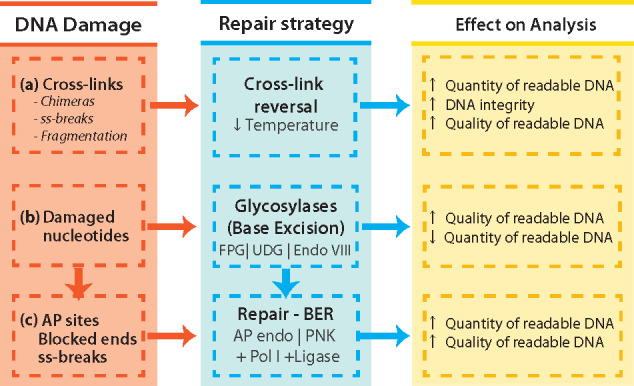
Summary of strategies applied for improving integrity, quantity and quality of bacterial DNA derived from FFPE specimens. (**a**) Exposure of DNA to denaturing temperatures (90°C) aids decrosslinking, but increases the rate of depurination and ss-break events that lead to the formation of ss-DNA regions known to favour the misincorporation of nucleotides (A – rule) or generate sequence chimeras. Therefore, milder decrosslinking reactions will reduce the rates of these occurrences. (**b**) The FFPE process damages DNA bases. The removal of damaged bases by glycosylases improves the quality of readable DNA by removing from the PCR pool damaged template that would otherwise lead to misincorporation of bases leading to SNPs. The product of either glycosylase treatments is AP sites (UDG) or 3′ blocked ends (FPG, Endo VIII) that block polymerase activity. (**c**) These blocking artefacts are repaired by either an AP endonuclease (AP sites Endo IV), leaving a 3′OH and 5′dRP, or a phosphokinase (3′P & T4 PNK), leaving a 3′OH and a 5′P. Only when ends are repaired (3′OH and 5′P/5′dRP), the DNA repair polymerase (Pol I) able to incorporate nucleotides that are subsequently sealed with a high fidelity DNA ligase (*E. coli* DNA ligase).

Having characterized the nature of FFPE-induced damage to bacterial DNA, an appropriate repair strategy was devised, as outlined in Fig. 2.

#### Optimization of decrosslinking

Crosslinks block polymerase processivity, reducing yields of PCR readable DNA [37]. Recently, it has been shown that 90°C decrosslinking incubation, reduces DNA sequence quality [20, 21]. For this reason, we aimed at investigating strategies that reduce heat exposure in order to find the optimal balance that improves the output DNA sequence quality without significantly affecting its yield.

#### Temperature

**Figure 3:**
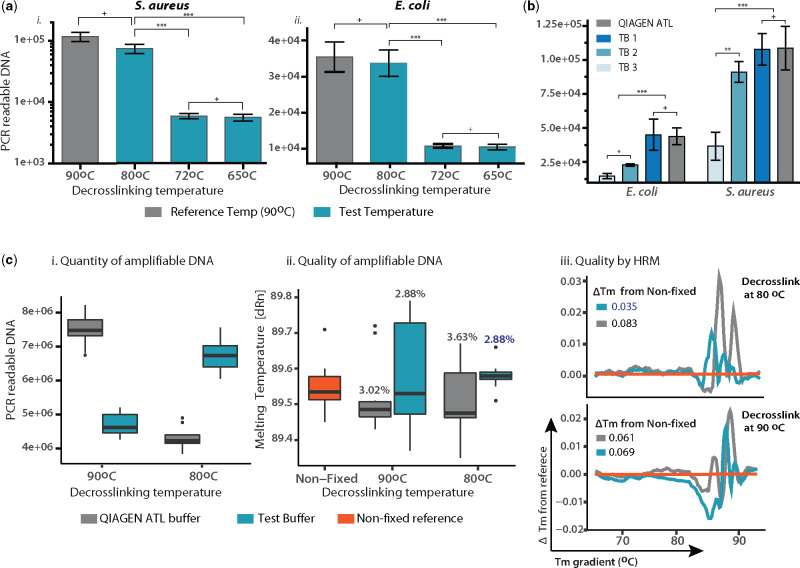
Optimizing a decrosslinking strategy. (**a**) Temperature. The bar plots show the recovery of 500 bp PCR readable DNA fragments after testing three cross-link reversal incubations (blue, for each bar *n* = 6) against a reference protocol (grey, *n* = 6). (**b**) Buffer. Three buffers were tested against the reference buffer at an 80°C × 1 h incubation. The amount of amplifiable DNA measured by qPCR of a 200 bp fragment in *E. coli* and *S. aureus* (for each bar *n* = 6). (**c**) Evaluating the optimized strategy. The quantity of amplifiable DNA (i) and the sequence quality of DNA (ii, iii) was assessed for a 500 bp DNA fragment. The performance of the optimized protocol (blue) was measured by comparing the reference protocol (grey). Box plot (i) shows the absolute quantity of amplifiable DNA from template DNA with normalized concentration (*n* = 6 for each box). In box plot (ii), the Tm of the tested conditions (*n* = 6 for each box) is compared with that of NF DNA (orange, *n* = 6). The Tm difference (ΔTm) between the test and NF DNA is indicated above each box. (iii) HRM plot – ΔTm of tests plotted against the Tm of NF sample (orange), with average ΔTm from NF shown above each plot (*n* = 6 for each line).

The effect of decrosslinking temperature on the yield of amplifiable DNA was investigated by quantitative PCR in DNA extracted from FFPE blocks loaded with *Staphylococcus aureus* (Fig. 3ai) and *E. coli* (Fig. 3aii), fixed for 24 h and stored for 3 months. Reactions were loaded with 10^6^ copies of template and incubated at 90°C for 1 h (reference protocol = industry standard mammalian DNA isolation from FFPE tissue), 80°C × 1 h, 72°C × 2 h or 65°C ×3 h. Compared with the reference 90°C protocol, no significant difference in amplification of PCR readable DNA was observed at 80°C for both bacteria (*P* > 0.05), while a ×4 (*E. coli*) and a ×10 (*S. aureus*) decrease in the amount of PCR readable DNA was evident at both 72°C and 65°C (*P* < 0.001). In this case, PCR amplification is indicative of the template fraction that was efficiently decrosslinked.

#### Buffers

The ability of three protein lysis buffers in setting reaction conditions (enthalpy disruption) that facilitate decrosslinking at 80°C were examined: Test Buffer 1 (TB1) – based upon the protein denaturing properties of chaotropic agents (GuHCl); Test Buffer 2 (TB2) – denaturing proteins with a reducing agents (DTT); Test Buffer 3 (TB3) – relying on the denaturing properties of an ionic detergent (sodium dodecyl sulphate). Decrosslinking with the three buffers was tested against the reference buffer (Buffer ATL, Qiagen FFPE Kit) at 80°C × 1 h. The effect of each buffer upon decrosslinking efficiency was assessed quantitatively by comparing the quantity of amplifiable DNA recovered after treatment. Contents of FFPE slides loaded with *E. coli* and *S. aureus* cells were suspended in each buffer (*n* = 6). Purified DNA was subjected to qPCR for amplification of a 200 bp fragment. TB1 and the reference displayed the highest yield (*P* > 0.05), significantly higher than TB2 (*P* < 0.05) and TB3 (*P* < 0.01); (Fig. 3b).

#### Evaluating DNA sequence quality of optimized strategy

The optimized strategy 1 h at 80°C in TB1 was tested against the standard protocol 1 h at 90°C in QIAGEN ATL Buffer for its capacity to decrosslink DNA, indicated by the yield of 500 bp PCR products (Fig. 3ci) and the sequence quality of the fragments yielded (Fig. 3cii and iii). This was tested in DNA sourced from FFPE blocks loaded with *E. coli* fixed for 48 h and stored for 1 year (representing maximum damage conditions). For quantitative analysis (Fig. 3ci), reactions were loaded with normalized DNA concentration. For qualitative analysis (Fig. 3cii and iii), reactions were loaded with 10^6^ amplifiable copies of the DNA fragments. Results are shown in Fig. 3ci, reflect those in Fig. 3b, with the new strategy (Buffer TB1 at 80°C), the yield of amplifiable DNA did not differ significantly from that of the reference protocol. However, the sequence quality of DNA recovered was improved with the new strategy. As it can be seen in Fig. 3cii, the Tm of samples treated with the new strategy was less variable and closer to that of paired-NF DNA, exhibiting a Tm difference [ΔTm (%)] of 2.88 (not significant), vs. 3.02 (*P* < 0.05) for the reference protocol. This was further confirmed with HRM (detailed in ‘Material and methods’ section), where aberrant profiles (from that of NF DNA) are indicative of randomly distributed sequence aberrations typical of FFPE DNA [49]. ΔTm plots in Fig. 3ciii, show that the ΔTm for samples decrosslinked with the new strategy [ΔTm (%) = 3.5] is significantly lower than that of the reference protocol (buffer ATL at 90°C) [ΔTm (%) = 6.1] (*P* < 0.05). This indicates that with the new strategy, without compromising DNA yields, the sequence quality of decrosslinked template is less damaged (resembles more NF DNA).

#### DNA glycosylases reduce sequence alterations in FFPE DNA

**Figure 4:**
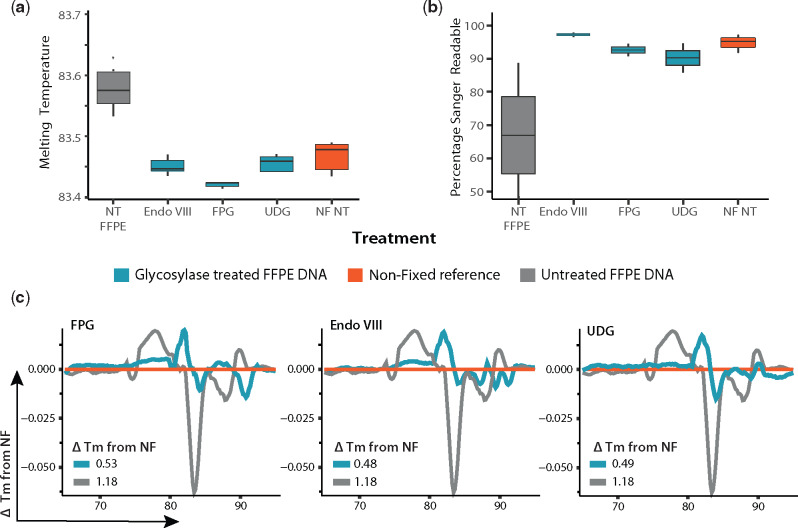
DNA glycosylases reduce sequence alterations in FFPE DNA. The reduction of sequence alterations in FFPE DNA (fixed for 48 h) by treatment with the selected glycosylases was confirmed by (**a**) Analysis of their Tm (*n* = 6 for each box). (**b**) Sanger sequencing readability (*n* = 3 for each box). (**c**) HRM (*n* = 6 for each line). In all tests performed, treatment with DNA glycosylases improved the amplifiable sequence quality. Grey: untreated FFPE samples. Orange: NF reference. Blue: glycosylases.

After examining their activity on FFPE DNA (Fig. 1c), the effect of treatment with DNA glycosylases on DNA sequence quality was assessed by: (i) Tm analysis, (ii) Sanger sequencing and (iii) HRM. For Tm analysis and HRM, all reactions were loaded with 1 × 10^6^ genome copies of DNA sourced from FFPE blocks loaded with *E. coli* and set to amplify 3 × 100 bp fragments (Fig. 4a and Supplementary Fig. S1). For all the regions analysed, the Tm of samples treated with all DNA glycosylases significantly changed from FFPE untreated samples (*P* < 0.001) and came closer to resemble that of the NF reference. This was further assessed by HRM, by comparing the melting profile of a 200 bp fragment (as explained in Fig. 3 and methods). As seen in Fig. 4c, the plotted ΔTm (from paired-NF) of glycosylases treated FFPE DNA was found to be much lower than that of untreated FFPE DNA. The same effect was evident with Sanger Sequencing (Fig. 4b), where treatment with all DNA glycosylases significantly improved (*P* < 0.001) the number of high-quality reads recovered, increasing the readability of DNA to levels no longer significantly different from NF DNA. Interestingly, samples treated with Endo VIII alone showed an improved sequence quality. Given that damage targeted by Endo VIII is PCR inhibitory, this might be indicative of activity in non-blocking lesions (Fapy-A), reflect PCR errors triggered by blocking lesions (jumping PCR) or be due to a reduction of *Taq* Polymerase fidelity (A rule and/or deletions) [52, 53].

#### *Development of an* in vitro *BER system*

For the *in vitro* reconstitution of the BER pathway, a suitable universal buffer was sought and tested by examining enzymatic activity for each enzyme (see Methods) and compared with activity in their recommended buffer (see Supplementary Fig. S2). Optimization of enzyme and co-factor quantity usage was then performed (Supplementary Tables S2–S4).

**Figure 5:**
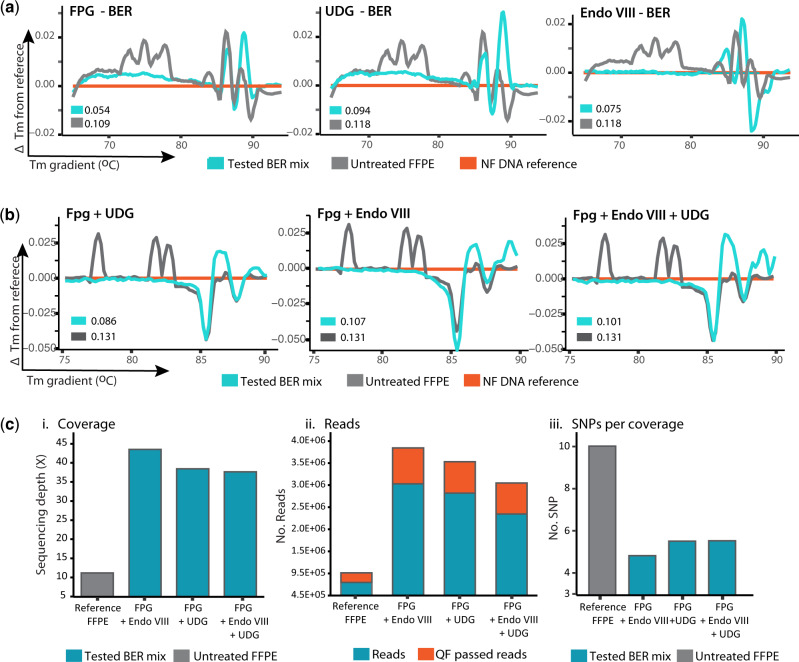
Reconstitution of BER pathway repairing FFPE DNA damage. (**a**) Single glycosylase BER. The BER pathway was reconstituted first as single pathways triggered by either UDG, FPG or Endo VIII. The efficiency of each system in correcting DNA damage was tested by HRM (*n* = 7 for each line). The more similar a DNA sequence is to the NF reference, the lower the difference in melting temperature (ΔTm closer to 0). FPG showed the highest efficiency in correcting FFPE DNA damage as evidenced by the lowest ΔTm of 0.054. (**b**) Multiple glycosylase BER. Mixes containing FPG show improved sequence quality as evidenced by reduced ΔTm vs. untreated. (**c**) WGS. To further confirm these results, six replicates treated with each mix were pooled (*n* = Σ6) and analysed by WGS. Data validated that all mixes improved the sequence (i) coverage, (ii) number of reads and QF passed reads and reduced the amount of SNPs (iii). The best performance in all cases was observed in the BER mix with FPG and Endo VIII.

First, the BER pathway was reconstituted for single repair pathways triggered by a single DNA glycosylase (UDG, FPG or Endo VIII), with units and enzymes listed in Supplementary Tables S3 and S4, and its performance was tested by HRM analysis. Figure 5a shows the HRM plots of DNA exposed to the BER pathway reconstituted for FPG, UDG or Endo VIII. As explained in methods, the more similar a DNA sequence is to the NF reference, the lower the difference in melting temperature (ΔTm closer to 0). As seen in Fig. 5a, exposure of DNA to each reconstituted BER pathway led to a reduction in ΔTm in FFPE DNA and an increase in the quantity of PCR readable template (Supplementary Fig. S3) suggesting a reduction in the frequency of sequence artefacts. The frequency of sequence artefacts observed after treatment was more effective for the FPG driven BER reaction, with a ∼50% decrease in ΔTm observed for untreated samples, this was followed by Endo VIII with a ∼31% reduction and finally UDG with a ∼14% decrease in the ΔTm. These results indicate that BER was reconstituted correctly and that these reconstituted pathways effectively corrected sequence artefacts without reducing the PCR readable template.

Subsequently, the reconstitution of a BER system able to target different types of DNA damage found in FFPE samples was addressed by mixing the pathways for the glycosylases treated in the system. Since FPG-BER (Fig. 5a) yielded the best results for single glycosylase–BER reactions, this enzyme was combined with ENDO VIII and/or UDG and their efficiency in reducing sequence artefacts tested by HRM. As shown in Fig. 5b, all combinations resulted in sequences with ΔTm lower than those of untreated FFPE DNA. The FPG + UDG mix showed the best performance at reducing the ΔTm (31%), followed by FPG + Endo VIII (18%). However, in terms of improving the PCR readability of a 500 bp fragment, FPG + Endo VIII (47% increase, *P* < 0.01) outperformed FPG + UDG (30% increase, *P* < 0.01), as measured by *Taq* qPCR. This effect was confirmed by high-fidelity qPCR (providing a more stringent discrimination of damaged and repaired sequence), where FPG + UDG showed a 20% increase and FPG + UDG only a 4% increase of amplifiable DNA (Supplementary Fig. S4). To confirm these results, a normalized DNA quantity from six replicates for each BER mix and six unrepaired samples were pooled into one (*n* = Σ6) and sent for analysis by WGS (Fig. 5c). At this level of resolution, it is evident that the repair mix with FPG + Endo VIII offered the highest improvements in sequence quality in terms of providing (i) a coverage ×4 higher than unrepaired, (ii) ×4 more total reads and quality filter (QF)-passed reads and (iii) a 50% reduction in the number of variants detected per sequence coverage. This repair mix was thus selected as the best repair mix for bacterial FFPE DNA.

#### Analysis of combined decrosslinking and BER treatment

**Figure 6:**
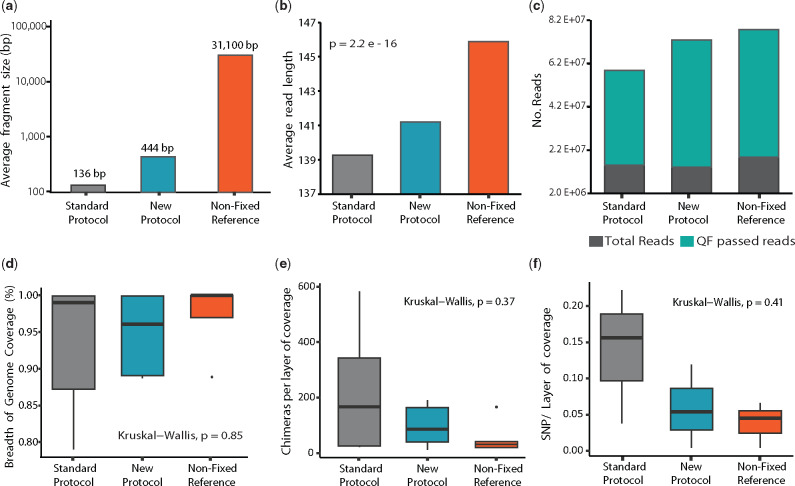
Combined protocol–bacterial DNA. Outputs of Bioanalyzer and WGS for bacterial FFPE DNA exposed to the combined treatment (blue, labelled as new protocol, Σ*n* = 6). This was compared with that obtained from six pooled paired-samples decrosslinked with the reference protocol and unrepaired (grey, labelled reference protocol, Σ*n* = 6) and that from DNA obtained from NF samples with the same bacterial and DNA content (orange, labelled NF, Σ*n* = 3). Improvement in DNA readability, sequence quality and integrity was measured by Integrity (fragment length): (**a**) fragment analyser (**b**) WGS. Readability: (**c**) Quantity of reads and filter pass reads per coverage. (**d**) Percentage of breath of genome coverage. Sequence quality: (**e**) number of chimeric reads per layer of coverage. (**f**) Number of SNPs per layer of coverage.

The sum of the above treatment strategies (decrosslinking and DNA repair) was tested by WGS in DNA sourced from FFPE blocks containing a mix of five bacterial strains, fixed for 48 h and stored for 2 months. DNA was decrosslinked at 80°C with TB1 (methods) and repaired with the FPG + Endo VIII–BER repair mix. The results of this were compared with those obtained from paired-samples treated with the reference protocol (decrosslinking at 90°C with QIAGEN ATL buffer, without DNA repair), and NF DNA obtained from equal cell contents. Experimental replicates were pooled (*n* = Σ6) and sent for WGS analysis. Results for this analysis are shown in Fig. 6 and Supplementary Fig. S5. The results obtained from exposing bacterial FFPE DNA to the proposed new protocol indicate that bacterial FFPE DNA treated with the proposed method shows an improvement in integrity, readability and sequence quality, as evidenced by (i) integrity [average fragment length (a, b)]: plotted in Fig. 6a are the average fragment lengths measured with a fragment analyser. Fragment length of DNA treated with the new protocol (444 bp) is ×3.3 longer than that treated with the reference protocol (136 bp). Importantly, this raises the average fragment length to that of fragments typically desired for 16S sequencing (460 bp). The same effect was observed in the length of fragments read by WGS, where fragment lengths were 2–3 bp longer on average (Fig. 6b). (ii) Readability: with the new protocol, the number of total reads and QF-pass reads per layer of coverage was increased by 24% and 34%, respectively, and the ratio of QF-passed to total reads increased by 8.4%. (iii) Sequence quality: this was measured in terms of number of sequence artefacts detected. The number of chimeric reads per coverage detected in samples treated with the new protocol was reduced by 57% (*P* = 0.37) (Fig. 6e). Similarly, the number of SNPs detected was reduced by 58% (*P* = 0.41) (Fig. 6f and Supplementary Fig. 5) in all strains tested. Despite the reduction in SNP’s being uniform across all strains tested, FFPE was found to produce a different SNP profile in Gram-positive vs. Gram-negative bacteria. As seen in Supplementary Fig. S6, a broad spectrum of SNPs was more proportionally abundant in FFPE Gram-negative *E. coli* when compared to the NF reference, while this was less pronounced in the Gram-positive *B. longum.* In these profiles, a reduction on SNPs derived from oxidative damage is observable in Gram-positive and Gram-negative bacteria; however, there is still a prevalence of SNPs-derived cytosine deamination.

**Figure 7:**
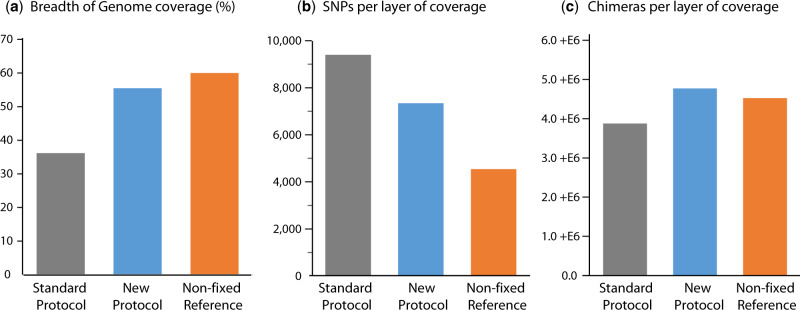
Sequence artefacts in mammalian DNA. Improvements in DNA quality and quantity were also obtained for mammalian DNA (4T1), for (**a**) percentage of breath of genome coverage, (**b**) number of SNPs per layer of coverage. However, a higher number of (**c**) sequence chimeras were detected.

Similar improvements in DNA quality and quantity to those shown in bacterial DNA were also obtained for the mammalian cell line used (4T1), where a 21% decrease in the amount of SNPs per layer of genome coverage and a 65% increase in the breadth of genome coverage was observed in the DNA treated with the proposed method (Fig. 7). Although these improvements were accompanied by a slight rise in chimeric sequences, the fact that this is seen in both the repaired samples and the NF reference indicates that this is likely a function of the increased reference genome coverage seen for these samples. All of these findings are coherent with results from quantitative PCR and Tm analysis. Although these improvements are not supported by statistical significance, given the considerable effect size, we are confident that this lack of significance is due to sample size alone. Altogether, the strategies proposed here were thoroughly investigated by PCR/sequencing, both individually and in combination. These results consistently indicate an improvement in the sequence integrity, readability and quality of readable bacterial FFPE DNA.

## Discussion

A plethora of studies has characterized FFPE-induced damage in human/mammalian DNA, where the abundance of DNA present in FFPE samples and presence of a well-characterized reference genome allow for high-quality reproducible research. To our knowledge, this is the first such study in prokaryotic DNA, where an understanding of effects of FFPE on DNA, and impact on downstream analyses is arguably even more important.

Our results show bacterial FFPE DNA to be a poor PCR template, with a log-fold reduction in the recovery of DNA fragments. This can be at least partially attributed to DNA fragmentation, since an inverse correlation between fragment size and PCR readability was shown (Fig. 1a), culminating in a log-fold reduction in recovery between 200 and 500 bp fragments. Crosslinks were found to be ubiquitous in FFPE bacterial DNA (Fig. 1b), and potentially more prevalent than in FFPE human DNA, based on previous research [12, 20]. Current decrosslinking protocols have been found to induce sequence alterations [21], and reducing heat exposure has been proposed to prevent this damage [20, 21]. Our results are in agreement with these hypotheses, as a reduction from 90°C (current protocols) to 80°C, showed a significant reduction in off-target effects, without compromising the decrosslinking efficiency. Here, we hypothesize that TB1 [containing 50 mM Tris-HCL (pH 8.0), 30 mM EDTA, 800 mM GuHCl, 0.5% Triton-X, 0.5% Tween-20] provided a reaction condition promoting decrosslinking at a lower temperature. This could be explained by a higher degree of protein denaturation, facilitated by GuHCl which interacts with multiple protein groups, including the backbone and hydrophobic and polar side chains. This is supported by GuHCl ability to increase the activity of Proteinase K and increase the torsional mobility of denatured proteins (at 1 M concentration) [54–56]. Furthermore, unlike SDS, chaotropes interacts with nucleic acids, altering their secondary and tertiary structure [57, 58]. In fact, 1 M concentrations of GuHCl have been shown to reduce the Tm of DNA by 13°C, and increase the stringency of its hybridization, promoting correct base pairing [59]. All of this would facilitate the exposure and hydrolysis of ubiquitous DPC [60, 61] and DNA–DNA complexes [62–64] at a lower temperature [65], reducing potential straining of the DNA structure and maintaining a high base paring fidelity. This could have been also assisted by other reaction conditions, such as pH and ionic strength [62, 66–68], Tris-HCl formaldehyde scavenger activity [61, 69] or possibly guanidinium–formaldehyde interactions, but this requires further investigation.

Treatment with glycosylases significantly reduces the appearance of sequence artefacts in FFPE DNA. Glycosylases generate blocked ends that are in most cases, unsuitable for amplification. This effect was confirmed in all glycosylases tested. Studies performed in human DNA have shown that cytosine deamination to uracil is the main source of sequence artefacts in FFPE DNA [12], although this has been controversial [14, 20, 21, 70]. Our data suggest that DNA damage found in bacterial FFPE DNA is primarily driven by oxidation and cytosine deamination, as evident in higher activity observed for FPG, Endo VIII and UDG. It is-known that oxidized products of cytosine can trigger its deamination [71]. While UDG repairs cytosine deamination and some of the oxidized deaminated lesions (5-OH dU), Endo VIII has a broader spectrum for these targets. Altogether, quantitative and qualitative analysis by qPCR (Fig. 5a and Supplementary Fig. S4) and sequencing (Fig. 5c) of samples treated with Endo VIII BER consistently yielded better results than UDG BER did, in terms of template readability and sequence fidelity. The same can be said for FPG + Endo VIII BER when compared to FPG + Endo VIII + UDG BER, despite some SNPs derived from cytosine deamination being evident in the repaired DNA profiles (Supplementary Fig. S6). While the HRM melting curve analysis provided a valuable guide, confirmation was provided by qPCR and sequencing data. After exhaustive comparisons of different approaches to the problem, the strategy found to be most effective involves decrosslinking using a chaotropic agent at 80°C, followed by DNA repair using a combination of formamidopyrimidine DNA glycosylase and Endonuclease VIII.

To conclude, the information generated here provides a better understating of FFPE-derived DNA damage, informing strategies for its repair. Here is also presented a thoroughly characterized method to address this damage. Given the increased activity in, and controversy surrounding, the field of low-biomass microbiome analysis, methods that improve the quality of microbiome studies (through sensitivity improvement or access to increased sample size) such as described here, are necessary. Given the paucity of published information on mammalian FFPE DNA repair, and none on bacterial repair, the strategy devised here provides compelling evidence to further pursue BER strategies to improve the sequencing quality of bacterial FFPE DNA and possibly mammalian FFPE DNA.

## Data availability

All sequencing data have been uploaded to the Sequence Read Archive: BioProject PRJNA627577.

## Supplementary data

Supplementary data is available at *Biology Methods and Protocols* online.

## Funding

This work was supported by the Irish Research Council [GOIPG/2016/475 to Y.F.B.], Science Foundation Ireland [12/RC/2273; 15/CDA/3630], the Health Research Board [MRCG2016-25] and Breakthrough Cancer Research.

## Conflict of interest

The authors declare that they have no competing interests.

## Supplementary Material

bpaa015_Supplementary_Data
