# Supplementary material for: Characterization of FFPE-induced bacterial DNA damage and development of a repair method
Source: Biol Methods Protoc. 2020 Jul 27;5(1):bpaa015. doi: 10.1093/biomethods/bpaa015 (PMC7548031; doi:10.1093/biomethods/bpaa015)
Supplement: bpaa015_Supplementary_Data [file bpaa015_supplementary_data.docx]

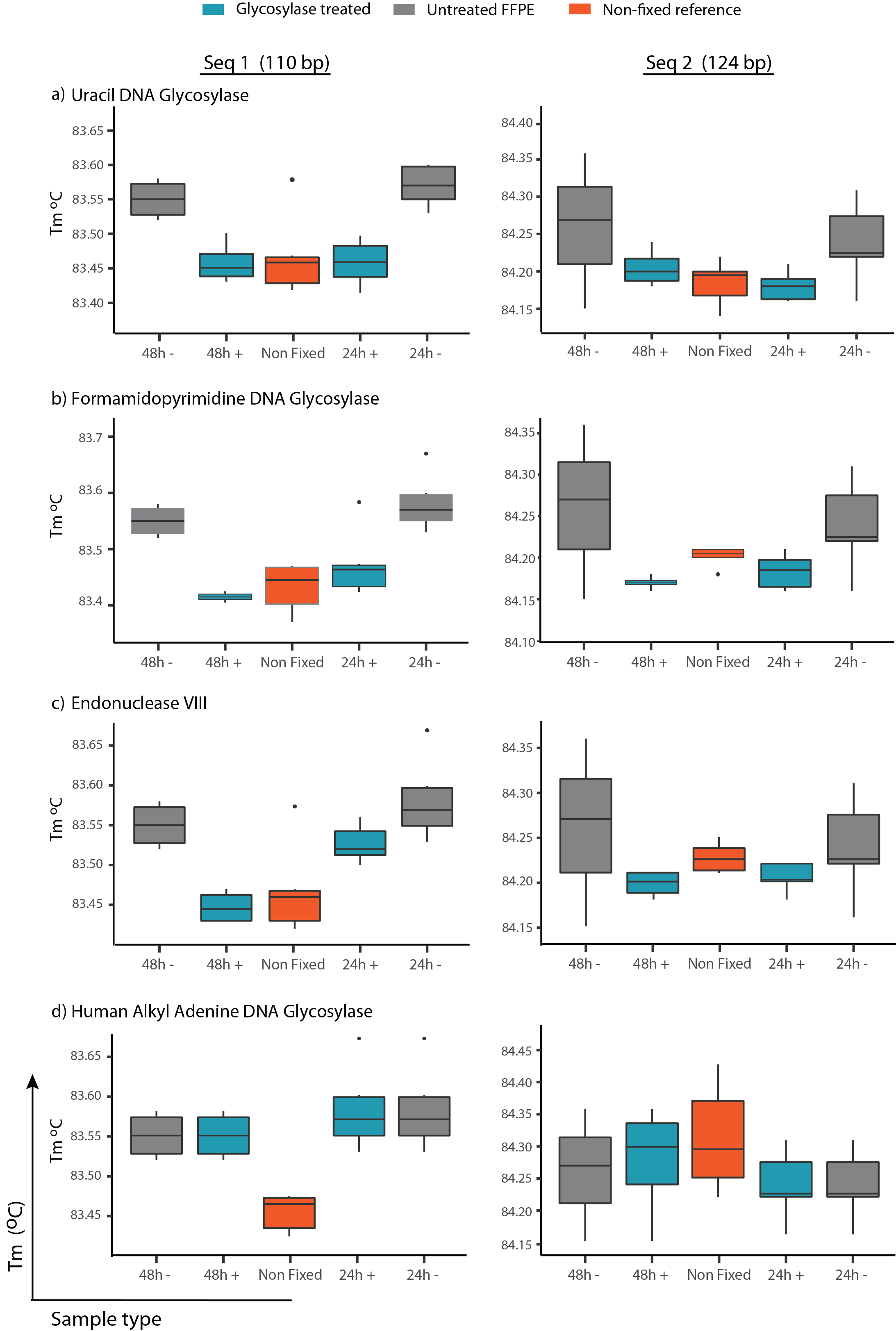


Supplementary Figure 1. Evaluating the effect of DNA glycosylases on bacterial FFPE DNA.

DNA purified from FFPE blocks loaded with E. coli fixed for 24h or 48h was pooled and equal quantities subjected to treatment with DNA glycosylases shown in plots. Tm analysis of 4 (≈ 100 bp) DNA sequences was performed on normalised quantities of amplifiable DNA. Shown here are the results for two sequences, wherein the melting temperature of fragments tested is compared between untreated DNA (grey, n = 12), NF DNA (orange, for each box n = 6) and glycosylase treated samples (blue, for each box n =6).


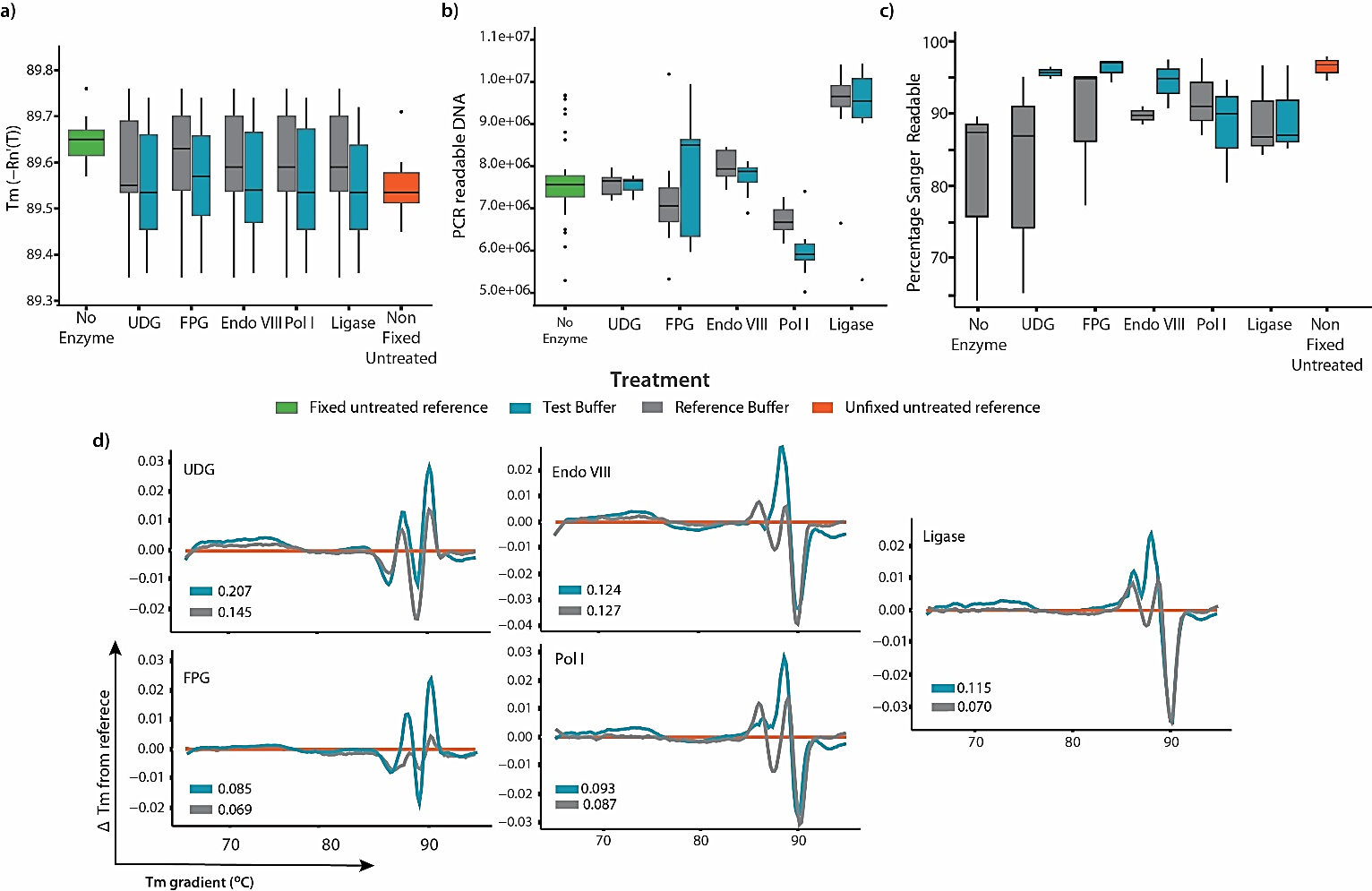


Supplementary Figure 2. DNA repair by BER system: Optimising a buffer.

A universal buffer (blue) allowing the reconstitution of the system was prepared and its influence on enzyme activity assessed by comparing its activity with the reference buffer (grey). This was analysed by: **a) Tm analysis** (each box n = 6)**, b) Recovery of amplifiable DNA** (each box n = 6)**, c) Sanger sequencing readability** (each box n = 3)**, d) HRM** (each box n = 6)**.** In all analysis the outputs of the enzyme activity using both buffers were comparable.


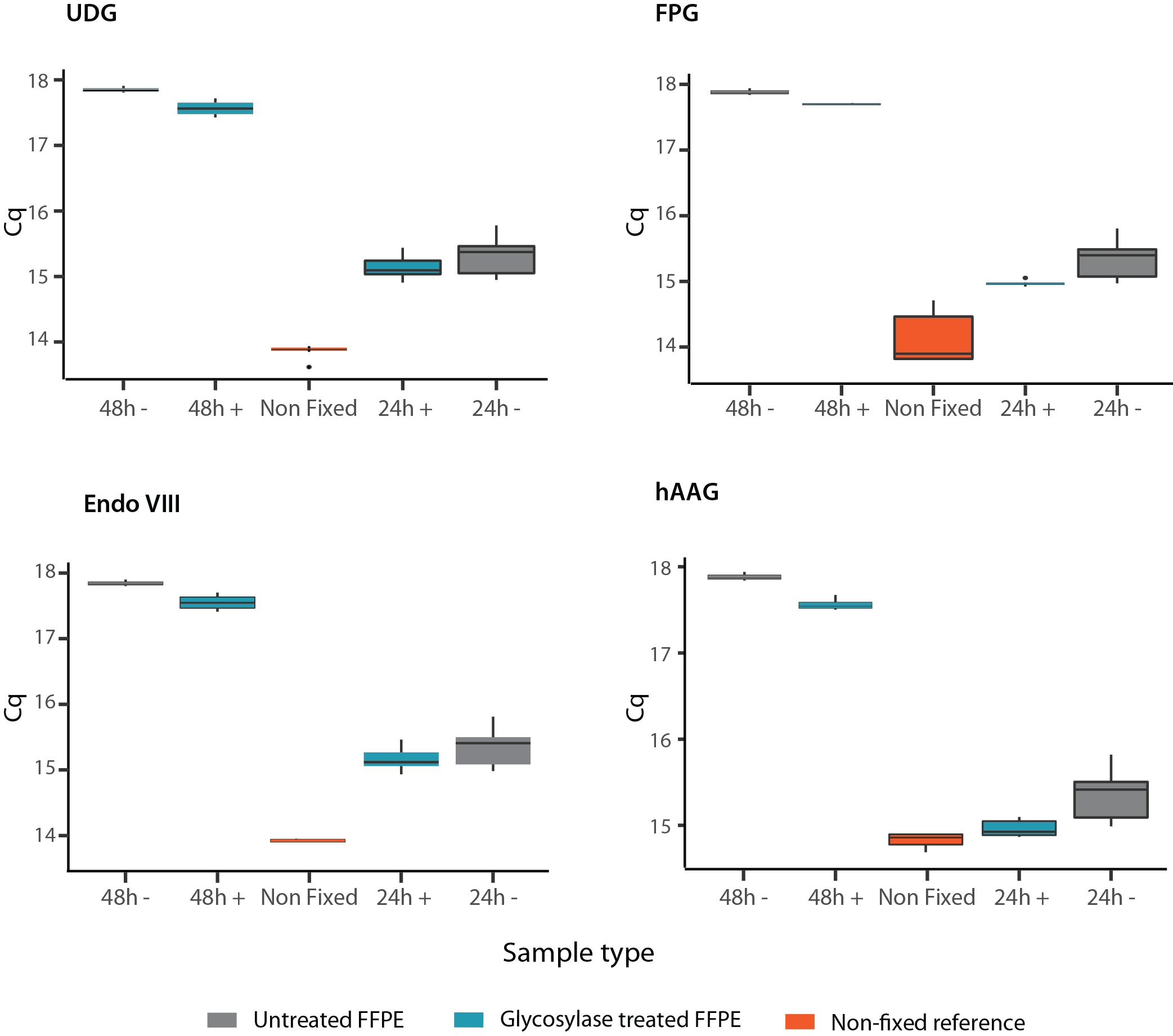


**Supplementary Figure 3. Quantitative analysis of treatment with glycosylases.** Box plot with average Cq obtained by qPCR after treatment with each glycosylase listed.


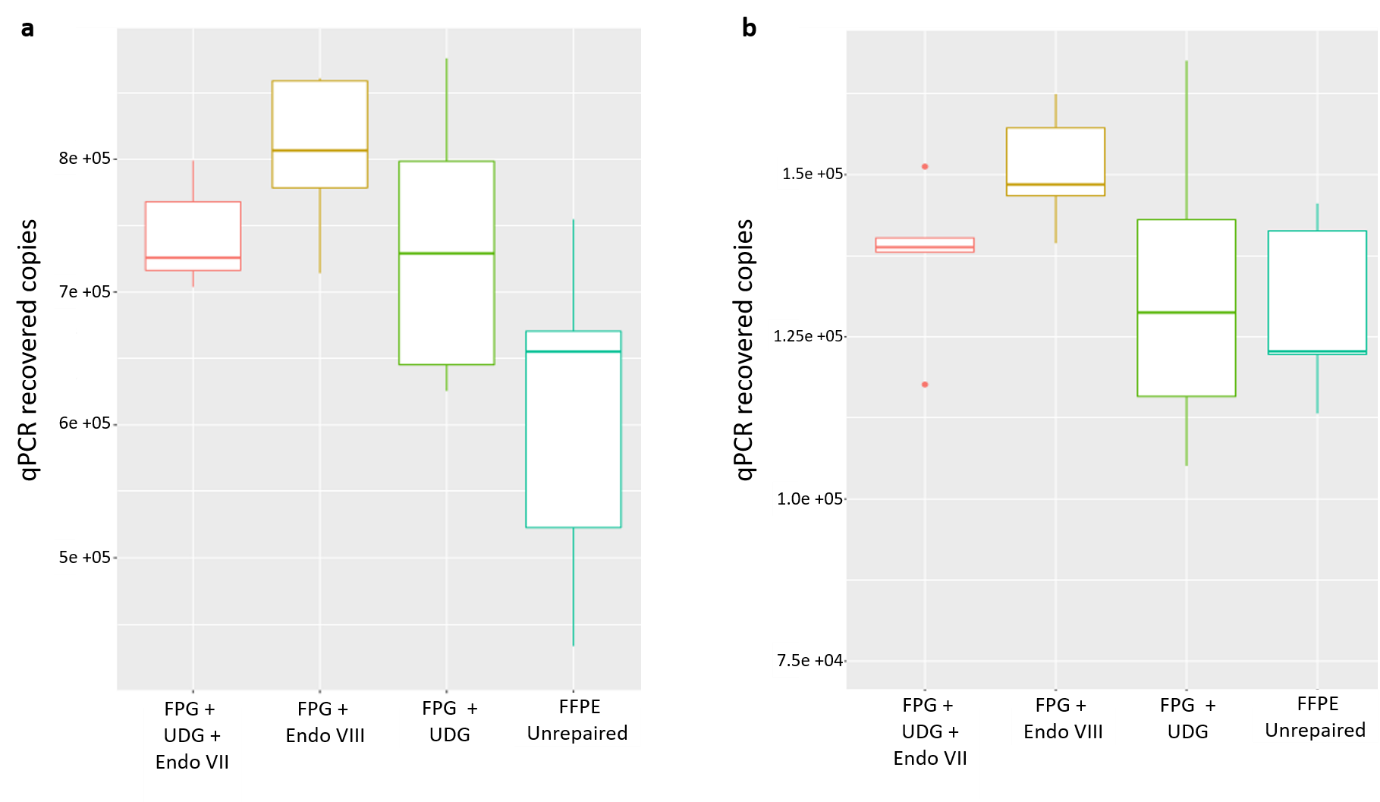


**Supplementary Figure 4.** **Quantitative analysis of treatment with single glycosylases BER mixes.** These enzyme mixes include: the DNA glycosylases being tested and the downstream repair enzymes (T4 PNK, Endo IV, Pol I and DNA Ligase) **(a)** Amplification with Taq Polymerase **(b)** Amplification with Q5.


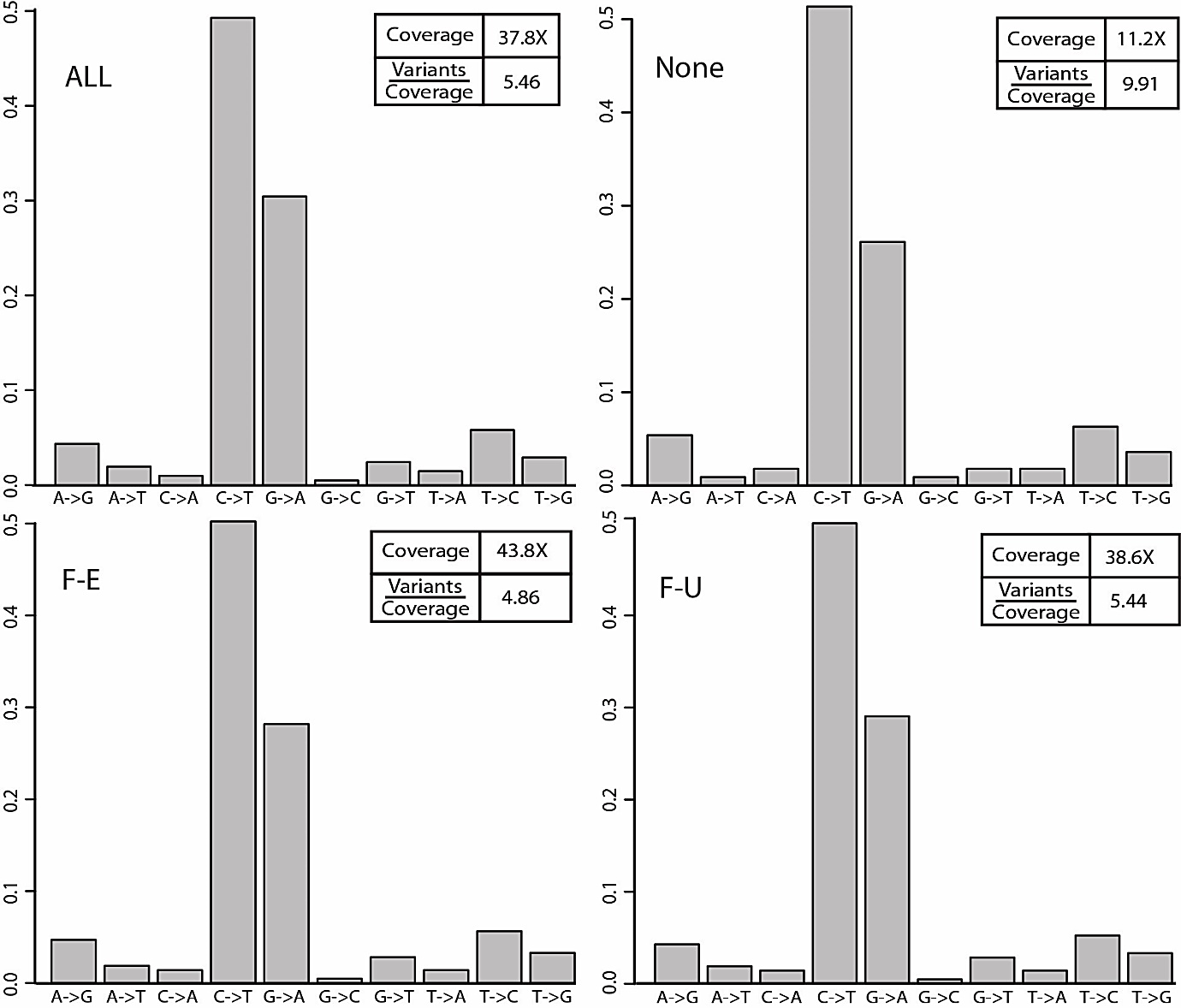


Supplementary Figure 5. SNP plots. Number of variants observed per repair strategy.


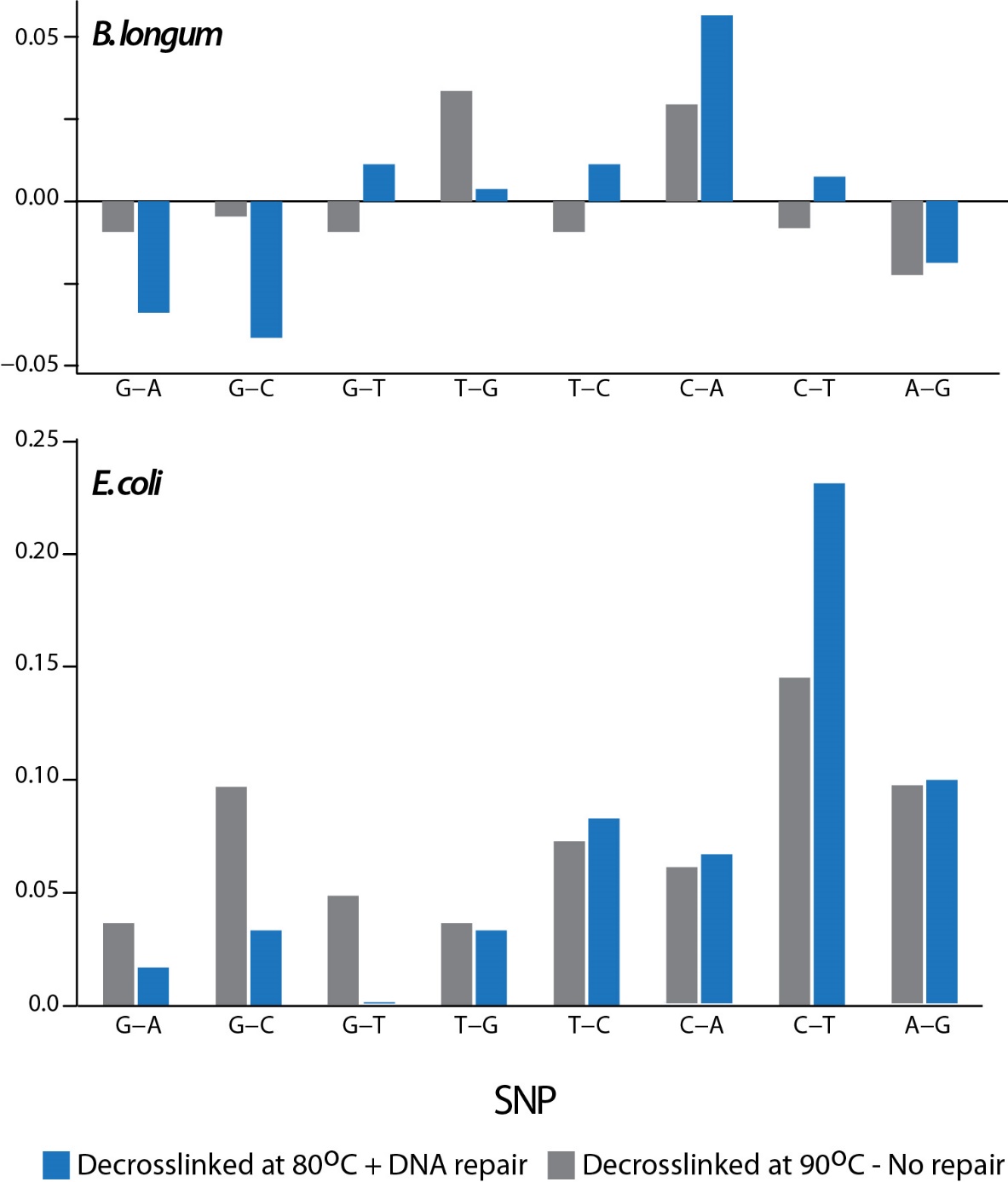


Supplementary Figure 6. SNP variation between E. coli (Gram-) and B. longum (Gram+).

**Supplementary Table 1.** Specifications of primers used for qPCR assays

| Strain/Cell line | Gene/ Accession No | Primer/Probe sequence | F/R^*^ | Product size (bp) |
| --- | --- | --- | --- | --- |
| *E coli* MG1655  [CP032667] | IS5-like element IS5 family transposase AYG17556.1 [CP032667: 230175-231191] | 5’TCA TTT GGT CCG CCC GAA AC | F | 525 |
|  |  | 5’CCA CCA TCA TTG AGG CAC CC | R |  |
|  |  | 5’GCC GAA CTG TCG CTT GAT GA | F | 217 |
|  |  | 5’ATT TGT CTC AGC CGA TGC CG | R |  |
|  |  | 5’TCG GCT GAG ACA AAT TGC TC | F | 110 |
|  |  | 5’GAT GCC AAG AGT GGC CTG | R |  |
|  |  | 5’ATG CCA AAG TGC CAC TGA T | F | 100 |
|  |  | 5’CCA CCA TCA TTG AGG CAC C | R |  |
|  |  | 5’CCC CTT GTA TCT GGC TTT CA | F | 116 |
|  |  | 5’AGA ACA AAA CGG CCA TCA AC | R |  |
| *Staphylococcus aureus* subsp*. aureus* str. Newman [CP023390.1] | Thermonuclease ATC67584.1  [CP023390.1:1359312-1359845] (1) | 5’ACG CCA GAA ACG GTG AAA C | F | 533 |
|  |  | 5’gac gta tta tta gcg aag cca tag agc | R |  |
|  |  | 5’CGC CTG TAC AAC CAT TTG GC | F | 182 |
|  |  | 5’tct agc aag tcc ctt ttc cac t | R |  |

*F= Forward primer, R = Reverse primer

**Supplementary Table 2.** Genomic data from *E. coli* used to calculate DNA glycosylases input.

| Genome size | 4,636,831 bp |
| --- | --- |
| Copy number per ng of DNA | 2.102 x 10^5^ |
| Moles per ng of DNA | 3.49 X 10 ^-19^ |
| Nucleotides per ng of DNA | 9.74 x 10^11^ |

**Supplementary Table 3.** Description of DNA glycosylases tested

| Enzyme | Damage targeted | Activity | Product | Units per ng of DNA in reaction | Excised bases | Inactivation |
| --- | --- | --- | --- | --- | --- | --- |
| **Uracil DNA Glycosylase** (Antarctic thermolabile)  (UDG) | **Deaminated cytosines:**  (dU, 5-OH-dU) (2) | Glycosylase | AP site | 0.004 | 7.20E+10 | 50^o^C  5 min |
| **Formamido-pyrimidine DNA glycosylase**  (FPG) | **Oxidised purines:**  (8-oxo-deoxypurines^1^,  Formamidopyrimidines^2^)  Oxidised pyrimidines^3^,  AP sites (3,4) | Glycosylase, β, δ - APlyase | 5’P and 3’ P* | 0.004 | 6.03E+09 | 60^o^C  10 min |
| **Endonuclease VIII**  (Endo VIII) | **Oxidised Pyrimidines:**  (dT and dU-Glycol, 5,6-dH-dT and dU, 5,6-diOH-dU and dC, 5-OH-6-H-dT and dU, 5-OH-dU and methylhydantoin)  Oxidised purine  (Fapy-dA) (5) | Glycosylase, β, δ - APlyase | 5’P and 3’ P | 0.008 | 9.13E+10 | 75^o^C  10 min |
| **Human Alkyl Adenine DNA Glycosylase**  (hAAG) | **Alkylated purines:**  3-me-dA, 7-me-dG, 1,N^6^-etheno-dA, hypoxanthine  Oxidised purines:  deoxy-dI and deoxy-xanthosine (6) | Glycosylase | AP site | 0.006 | 2.97E+10 | 65^o^C  10 min |

***** P = phosphates; dT: deoxy-thymine; dA: deoxy-adenine; dC: deoxy-cytosine; dU: deoxy-uracil; dI: deoxy-Inosine; OH: Hydroxy; diOH: dihydroxyl me: methyl, dH: dihydroxyl

^1^ 8-oxodeoxypurines: 8-oxo-dG, 8-oxo-dA, 8-oxo-dNebularine, and 8-oxo-dInosine

^2^Formamidopyrimidines: fapy-dG, fapy-dA, and me-fapy-dG

^3^Oxidised pyrimidines: 5-hydroxy-deoxycytosine and 5-hydroxy-deoxyuridine.

**Supplementary Table 4.** Description of downstream lesion repair enzymes.

| **Enzyme** | **For**  **Glycosylase** | **Activity** | **Product** | **Units per ng of DNA in reaction** | **Repaired**  **bases/ends** | **Cofactor** |
| --- | --- | --- | --- | --- | --- | --- |
| **Endonuclease IV**  ( Endo IV) | UDG, hAAG | Removes  AP sites (7,8) | 3’OH and 5’dRP* | 0.01 | 4.52E+09 | - |
| **T4 Polynucleotide DNA Kinase** (PNK) | FPG, Endo VIII | Removes  3’ Phosphates (9) | 3’OH and 5’P* | 0.017 | 1.25E+10 | DTT  (5 mM) |
| **DNA**  **Polymerase I**  (Pol I) | All | 5’-3’ Exonuclease removes 5’dRP and fills nicks (7,8) | Nick translation & nucleotide incorporation | 0.015 | 9.74E+16 | dNTPs  (33 µM) |
| **E coli DNA ligase** | All | Gap sealing (7,8) | PD bond between 5´P and 3’OH | 0.025 | 7.23E+09 | NAD+  (50 µM) |

***** P = phosphates, dRP = deoxyribose phosphate, PD = phosphodiester bond

**REFERENCES**

1. Madison, B.M. and Baselski, V.S. (1983) Rapid identification of Staphylococcus aureus in blood cultures by thermonuclease testing. *J Clin Microbiol*, **18**, 722-724.

2. Krokan, H.E., Drabløs, F. and Slupphaug, G. (2002) Uracil in DNA – occurrence, consequences and repair. *Oncogene*, **21**, 8935-8948.

3. Hatahet, Z., Kow, Y.W., Purmal, A.A., Cunningham, R.P. and Wallace, S.S. (1994) New substrates for old enzymes. 5-Hydroxy-2'-deoxycytidine and 5-hydroxy-2'-deoxyuridine are substrates for Escherichia coli endonuclease III and formamidopyrimidine DNA N-glycosylase, while 5-hydroxy-2'-deoxyuridine is a substrate for uracil DNA N-glycosylase. *J Biol Chem*, **269**, 18814-18820.

4. Tchou, J., Bodepudi, V., Shibutani, S., Antoshechkin, I., Miller, J., Grollman, A.P. and Johnson, F. (1994) Substrate specificity of Fpg protein. Recognition and cleavage of oxidatively damaged DNA. *J Biol Chem*, **269**, 15318-15324.

5. Dizdaroglu, M., Burgess, S.M., Jaruga, P., Hazra, T.K., Rodriguez, H. and Lloyd, R.S. (2001) Substrate Specificity and Excision Kinetics of Escherichia coli Endonuclease VIII (Nei) for Modified Bases in DNA Damaged by Free Radicals. *Biochemistry*, **40**, 12150-12156.

6. Lee, C.-Y.I., Delaney, J.C., Kartalou, M., Lingaraju, G.M., Maor-Shoshani, A., Essigmann, J.M. and Samson, L.D. (2009) Recognition and processing of a new repertoire of DNA substrates by human 3-methyladenine DNA glycosylase (AAG). *Biochemistry*, **48**, 1850-1861.

7. Krwawicz, J., Arczewska, K.D., Speina, E., Maciejewska, A. and Grzesiuk, E. (2007) Bacterial DNA repair genes and their eukaryotic homologues: 1. Mutations in genes involved in base excision repair (BER) and DNA-end processors and their implication in mutagenesis and human disease. *Acta Biochim Pol*, **54**, 413-434.

8. Dianov, G. and Lindahl, T. (1994) Reconstitution of the DNA base excisionrepair pathway. *Current Biology*, **4**, 1069-1076.

9. Dobson, C.J. and Allinson, S.L. (2006) The phosphatase activity of mammalian polynucleotide kinase takes precedence over its kinase activity in repair of single strand breaks. *Nucleic acids research*, **34**, 2230-2237.
